# Association of a Diagnosis of Ductal Carcinoma In Situ With Death From Breast Cancer

**DOI:** 10.1001/jamanetworkopen.2020.17124

**Published:** 2020-09-16

**Authors:** Vasily Giannakeas, Victoria Sopik, Steven A. Narod

**Affiliations:** 1 Women’s College Research Institute, Toronto, Ontario, Canada; 2 Dalla Lana School of Public Health, University of Toronto, Toronto, Ontario, Canada; 3 ICES, Toronto, Ontario, Canada; 4 Institute of Medical Science, University of Toronto, Toronto, Ontario, Canada

## Abstract

**Importance:**

It is not clear to what extent a diagnosis of ductal carcinoma in situ (DCIS) impacts a woman’s lifetime risk of dying of breast cancer. Under ideal circumstances, treatment will eliminate the risk of invasive ipsilateral recurrence and prevent subsequent mortality from breast cancer. The risk of dying of breast cancer after a diagnosis of DCIS had not been compared with that of women without cancer in the general population.

**Objective:**

To determine the risk of death from breast cancer in a large cohort of patients treated for DCIS and to compare the risk with that of women in the general population.

**Design, Setting, and Participants:**

This cohort study included data for women who had first primary DCIS diagnosed between 1995 and 2014 from the Surveillance, Epidemiology and End Results (SEER) registries database. Women with DCIS underwent surgical treatment, and approximately half also received radiotherapy. These women were followed from the date of DCIS diagnosis until death from breast cancer or date of last follow-up. Women in the general population without breast cancer were analyzed as controls. Follow-up information was available up to December 2016. The data were analyzed in March 2020.

**Exposures:**

Patients with DCIS who underwent surgical treatment.

**Main Outcomes and Measures:**

Breast cancer death was the main outcome. Standardized mortality ratios were estimated by comparing deaths from breast cancer among women diagnosed with DCIS with expected deaths from breast cancer among women in the general population who did not have cancer. Expected probability of death from breast cancer in the general population was calculated by an incidence-based mortality approach using standardized SEER-based incidence and case-fatality rates. Probability of breast cancer death was estimated based on the assumption that a cancer-free control was cancer free on the date the woman with DCIS was diagnosed and was studied until the end of follow-up.

**Results:**

A total of 144 524 women diagnosed with first primary DCIS were included (mean [SD] age at diagnosis, 57.4 [11.0] years). There were 1540 deaths from breast cancer in the cohort. Based on SEER-based incidence and case-fatality rates, 458 breast cancer deaths were expected in an equivalent number of cancer-free women from the general population with equal follow-up. The standardized mortality ratio for death from breast cancer among women with DCIS was 3.36 (95% CI, 3.20-3.53). The elevated risk of death persisted more than 15 years after diagnosis.

**Conclusions and Relevance:**

In the population studied, the risk of dying of breast cancer was increased 3-fold after a diagnosis of DCIS. This suggests that our current treatment focus on preventing invasive recurrence is insufficient to eliminate all deaths from breast cancer after DCIS.

## Key Points

**Question:**

Is ductal carcinoma in situ (DCIS) associated with lifetime risk of dying of breast cancer?

**Findings:**

In this cohort study of 144 524 women treated for DCIS, 1540 women died of breast cancer, while based on national incidence and case-fatality rates, the expected number of deaths from breast cancer was 458. Women with DCIS had a 3-fold increased risk of dying of breast cancer compared with women without DCIS.

**Meaning:**

These findings suggest that the current treatment of DCIS does not eliminate the risk of breast cancer mortality.

## Teaser

This cohort study examines risk of dying of breast cancer among women with ductal carcinoma in situ compared with women in the general population.

## Introduction

Ductal carcinoma in situ (DCIS) refers to the histological appearance of cancer cells within the breast ductule/lobule without evidence of cancer cells present beyond the basement membrane.^1^ DCIS is generally identified in asymptomatic women who undergo screening mammography.^2^ Approximately 3% of women with DCIS will die of breast cancer within 20 years; however, the risk is greater for young women and Black women.^3^

It is believed that DCIS in itself is not life-threatening but can progress to invasive breast cancer. The 2 goals of treatment are to prevent invasive ipsilateral recurrence and to prevent death from breast cancer. It is proposed that death from breast cancer following a diagnosis of DCIS is a consequence of the intervening invasive breast cancer and that control of invasive ipsilateral recurrence is a critical step in preventing death from breast cancer. Treatment options include surgical procedures (ie, lumpectomy or mastectomy), radiation, and hormonal therapy.

Under ideal circumstances, all local invasive recurrences would be prevented, as would all deaths from breast cancer following DCIS. If the absolute risk of dying of breast cancer for women with DCIS fell below that of the general population, this would be a strong argument in favor of current treatment protocols for DCIS and an endorsement of screening in general. On the other hand, if the mortality rate from breast cancer among women with DCIS was found to be much higher than that of the general population, this would require reevaluation of the underlying 2-step model as well as reconsideration of the current treatment paradigm. In this study using Surveillance, Epidemiology and End Results (SEER) registry data, we estimated the risk of dying of breast cancer after a diagnosis of DCIS and compared this with the risk of a woman in the general population who is breast cancer–free at the time of follow-up.

## Methods

This cohort study was deemed exempt from research ethics board approval and informed consent by Women’s College Hospital because study participants were ascertained through a deidentified and publicly available database. Our study adheres to the Strengthening the Reporting of Observational Studies in Epidemiology (STROBE) reporting guideline.

We used the SEER*Stat version 8.3.6 to conduct a case-listing session and retrieved all cases of first primary DCIS (stage 0) diagnosed between 1995 and 2014 in the SEER18 registries research database (November 2018 submission). We selected all cases with the American Joint Committee on Cancer primary tumor classification *Tis* (ie, carcinoma in situ; no evidence of an invasive component). We excluded cases with microinvasion (7.7% of DCIS cases). Among the cases classified as Tis, we excluded those associated with lobular carcinoma in situ, nonepithelial histological presentations, Paget disease of the nipple, or diffuse DCIS. We also excluded cases with unknown laterality or no surgical intervention on the primary tumor. We excluded cases diagnosed in women younger than 25 years or aged 80 years or older.

For each case, we retrieved information on the year of breast cancer diagnosis, age at diagnosis, median annual household income, race/ethnicity, tumor laterality, tumor size, tumor grade, estrogen receptor (ER) status, progesterone receptor (PR) status, use of radiotherapy, and type of surgical treatment. Measures in the follow-up period included ipsilateral invasive recurrence, contralateral invasive breast cancer, a new non-breast primary cancer, death, and cause of death. We assessed the vital status at the time of last follow-up. We extracted the information on survival time from the variable *survival time months*. The SEER*Stat program estimates survival time by subtracting the date of diagnosis from the date of last contact (the study cutoff). The study cut-off date was December 31, 2016. The data were analyzed in March 2020.

Women with DCIS were followed from DCIS diagnosis until death from breast cancer, death from another cause, loss to follow-up, 20 years after DCIS diagnosis, or December 31, 2016, whichever occurred first. We counted the observed number of deaths from breast cancer for the cohort.

### Statistical Analysis

To estimate the expected number of deaths from breast cancer in the cohort we calculated the expected probability of death from breast cancer for each of the women with DCIS under the assumption that she was cancer-free at the time of DCIS. That is, if a woman received a diagnosis of DCIS at age 50 years and was followed until age 60 years, we asked: what is the probability that a women without cancer at age 50 years would develop and die of breast cancer by age 60 years? We calculated the expected probability of death from breast cancer using an incidence-based mortality approach. We acquired age- and calendar year–specific breast cancer incidence rates from the SEER incidence registry between 1995 and 2014. We derived age- and calendar year–specific breast cancer mortality rates by year of follow-up among women diagnosed with a first primary invasive breast cancer (stage I to IV) between 1995 and 2014. Calendar year–specific values were defined based on 4 equally sized calendar year intervals: 1995 to 1999, 2000 to 2004, 2005 to 2009, and 2010 to 2014.

For each woman in the DCIS cohort, we calculated an expected probability of death from breast cancer for a woman in the general population who was cancer-free and with an equal follow-up time. To calculate the expected probability of death within a given interval we multiplied the age- and year-specific incidence rates of breast cancer by the breast cancer mortality rates, which are conditional on age at diagnosis, year of diagnosis, and time since diagnosis. To estimate the total expected number of breast cancer deaths in the cohort we summed the individual probabilities of death from breast cancer for each of the women in the cohort.

We compared the observed number of deaths to the expected number of deaths as a standardized mortality ratio (SMR) for the entire cohort and for subgroups defined by age, race/ethnicity, and surgical treatment. We performed bootstrap sampling (1000 sampling iterations) to obtain 95% CIs for the SMR values.

Data were analyzed using SAS statistical software version 9.4 (SAS Institute). *P* values were 2-sided, and statistical significance was set at *P* < .05.

## Results

Among the cohort of 144 524 women with DCIS (mean [SD] age at diagnosis, 57.4 [11.0] years), 1540 women died of breast cancer (Table 1). The earliest case of DCIS was recorded in 1995, and the most recent death was recorded in 2016. The 144 524 cases contributed a total of 1 326 075 person-years of follow up. The mean (SD) period of follow up was 9.2 (4.9) years (range, 0.1-20.0 years). There were 4502 (3.1%) ipsilateral invasive recurrence events in the follow-up period, resulting in a 20-year actuarial risk of 13.9%. There were 5527 (3.8%) contralateral invasive breast cancer events in the follow-up period, resulting in a 20-year actuarial risk of 11.3%. The 20-year actuarial risk of breast cancer death among women with DCIS was 3.3%.

**Table 1.** Characteristics of Women With DCIS

Variable	Women, No. (%) (N = 144 524)
Year of diagnosis	
Mean (SD) [95% CI]	2006.3 (5.2) [2006.3-2006.3]
Median (IQR) [range]	2007.0 (2002.0-2011.0) [1995.0-2014.0]
1995-1999	15 797 (10.9)
2000-2004	38 201 (26.4)
2005-2009	43 426 (30.0)
2010-2014	47 100 (32.6)
Age at diagnosis, y	
Mean (SD) [95% CI]	57.4 (11.0) [57.4-57.5]
Median (IQR) [range]	57.0 (49.0-66.0) [25.0-79.0]
<40	5146 (3.6)
40-49	33 966 (23.5)
50-59	43 737 (30.3)
60-69	37 432 (25.9)
70-79	24 243 (16.8)
Race/ethnicity	
White	112 539 (77.9)
Black	15 415 (10.7)
East Asian	6344 (4.4)
Southeast Asian	5664 (3.9)
Other or unknown	4562 (3.2)
Income, $	
Mean (SD) [95% CI]	48 812 (11 380) [48 754-48 871]
Median (IQR) [range]	47 070 (42 070-55 950) [15 810-79 890]
<40 000	26 729 (18.5)
40 000-45 000	35 277 (24.4)
45 000-50 000	23 098 (16.0)
>50 000	59 407 (41.1)
Missing	13 (<0.1)
Histological presentation	
Comedonecrosis	17 246 (11.9)
Cribriform	12 799 (8.9)
Intraductal, solid type	68 449 (47.4)
Other ductal, NOS	38 048 (26.3)
Papillary	7982 (5.5)
Tumor grade	
I	16 539 (11.4)
II	48 852 (33.8)
III/IV	53 924 (37.3)
Unknown	25 209 (17.4)
Tumor size, cm	
Mean (SD) [95% CI]	1.7 (2.1) [1.7-1.7]
Median (IQR) [range]	1.1 (0.6-2.0) [0.1-98.8]
<1	43 152 (29.9)
1-2	28 877 (20.0)
2-3	12 561 (8.7)
3-5	9279 (6.4)
>5	6876 (4.8)
Unknown	43 779 (30.3)
ER status	
Positive	76 611 (53.0)
Negative	13 730 (9.5)
Unknown	54 183 (37.5)
PR status	
Positive	63 623 (44.0)
Negative	21 405 (14.8)
Unknown	59 496 (41.2)
Surgical treatment	
Lumpectomy	96 806 (67.0)
Mastectomy	38 870 (26.9)
Unknown (diagnosis prior to 1998)	8507 (5.9)
Unknown	341 (0.2)
Radiotherapy	
No	74 441 (51.5)
Yes	68 118 (47.1)
Unknown	1965 (1.4)
Follow-up time, y	
Mean (SD) [95% CI]	9.2 (4.9) [9.1-9.2]
Median (IQR) [range]	8.7 (5.1-13.0) [0.1-20.0]
Death in follow-up	
No	128 991 (89.3)
Yes	15 533 (10.7)
Death from breast cancer	
No	142 984 (98.9)
Yes	1540 (1.1)
Death from other cause	
No	130 531 (90.3)
Yes	13 993 (9.7)
Vital status	
Alive	128 991 (89.3)
Death from breast	1540 (1.1)
Death from other cancer	3874 (2.7)
Death from heart disease	3880 (2.7)
Death from other diseases	3619 (2.5)
Unknown cause of death	2620 (1.8)

Abbreviations: DCIS, ductal carcinoma in situ; ER, estrogen receptor; IQR, interquartile range; NOS, not otherwise specified; PR, progesterone receptor.

Based on national incidence and case-fatality rates, the expected number of deaths from breast cancer in the cancer-free cohort was 458 deaths. This comparison assumes that each woman was cancer-free at the time of DCIS diagnosis in the cohort of women with DCIS. The SMR for death from breast cancer given a diagnosis of DCIS was 3.36 (95% CI, 3.20-3.53) (Table 2).

**Table 2.** Subgroup SMR Analysis

Variable	Patients, No. (%)	Person-years, No.	Breast cancer deaths, No.		SMR (95% CI)
			Observed	Expected	
All patients	144 524 (100)	1 326 075.4	1540	458.6	3.36 (3.20-3.53)
Year of diagnosis					
1995-1999	15 797 (10.9)	257 386.8	426	129.5	3.29 (3.00-3.61)
2000-2004	38 201 (26.4)	494 929.3	682	196.0	3.48 (3.22-3.76)
2005-2009	43 426 (30.0)	379 730.8	343	104.0	3.30 (2.96-3.64)
2010-2014	47 100 (32.6)	194 028.6	89	29.1	3.05 (2.47-3.74)
Age at diagnosis, y					
<40	5146 (3.6)	53 117.4	98	8.2	11.95 (9.66-14.39)
40-49	33 966 (23.5)	330 027.0	315	75.9	4.15 (3.73-4.59)
50-59	43 737 (30.3)	414 042.8	383	135.9	2.82 (2.54-3.09)
60-69	37 432 (25.9)	329 418.2	356	134.3	2.65 (2.39-2.92)
70-79	24 243 (16.8)	199 470.0	388	104.3	3.72 (3.35-4.11)
Race/ethnicity					
White	112 539 (77.9)	1 049 781.8	1121	369.6	3.03 (2.86-3.21)
Black	15 415 (10.7)	131 272.5	319	42.2	7.56 (6.76-8.42)
East Asian	6344 (4.4)	59 965.3	40	21.2	1.89 (1.36-2.49)
Southeast Asian	5664 (3.9)	47 916.1	35	14.6	2.40 (1.65-3.31)
Other or unknown	4562 (3.2)	37 139.8	25	11.1	2.25 (1.41-3.13)
Black race, by age group, y					
<40	692 (4.5)	6856.0	22	0.99	22.19 (13.86-32.85)
40-49	3450 (22.4)	32 079.7	75	7.02	10.68 (8.29-13.36)
50-59	4736 (30.7)	41 260.1	94	12.72	7.39 (5.89-8.92)
60-69	4118 (26.7)	33 070.8	64	12.68	5.05 (3.84-6.28)
70-79	2419 (15.7)	18 006.0	64	8.76	7.30 (5.59-9.19)
Income, $					
<40 000	26 729 (18.5)	231 151.1	321	78.1	4.11 (3.64-4.57)
40 000-45 000	35 277 (24.4)	321 855.9	458	113.2	4.05 (3.68-4.44)
45 000-50 000	23 098 (16.0)	216 038.8	238	75.7	3.15 (2.75-3.56)
>50 000	59 407 (41.1)	556 949.6	523	191.6	2.73 (2.50-2.97)
Tumor grade					
I	16 539 (11.4)	144 552.1	112	50.3	2.23 (1.82-2.67)
II	48 852 (33.8)	419 251.2	405	137.0	2.96 (2.67-3.24)
III-IV	53 924 (37.3)	477 348.4	598	155.6	3.84 (3.52-4.14)
Unknown	25 209 (17.4)	284 923.8	425	115.8	3.67 (3.34-4.04)
Histological presentation					
Comedonecrosis	17 246 (11.9)	178 803.8	289	68.7	4.21 (3.74-4.73)
Cribriform	12 799 (8.9)	107 254.3	79	34.4	2.29 (1.83-2.81)
Intraductal, solid type	68 449 (47.4)	674 710.6	822	244.1	3.37 (3.14-3.59)
Other ductal, NOS	38 048 (26.3)	283 538.3	253	80.0	3.16 (2.74-3.55)
Papillary	7982 (5.5)	81 768.5	97	31.4	3.09 (2.48-3.68)
Tumor size, cm					
<1	43 152 (29.9)	373 991.8	306	125.3	2.44 (2.17-2.75)
1-2	28 877 (20.0)	253 990.3	284	87.2	3.26 (2.89-3.67)
2-3	12 561 (8.7)	105 549.8	153	34.0	4.50 (3.79-5.24)
3-5	9279 (6.4)	76 579.5	88	23.6	3.72 (2.95-4.55)
>5	6876 (4.8)	55 555.2	94	16.1	5.83 (4.78-7.07)
Unknown	43 779 (30.3)	460 408.8	615	172.3	3.57 (3.30-3.85)
Subtype					
ER and PR positive	62 774 (43.4)	433 132.5	335	112.9	2.97 (2.64-3.26)
ER positive/PR negative	9156 (6.3)	66 889.2	74	19.0	3.90 (3.06-4.92)
ER negative and PR positive	803 (0.6)	6983.1	17	2.2	7.69 (4.34-11.89)
ER and PR negative	12 236 (8.5)	93 144.0	134	27.5	4.87 (4.06-5.74)
Unknown	59 555 (41.2)	725 926.7	980	297.0	3.30 (3.10-3.51)
Treatment comparison					
Lumpectomy alone	31 441 (33.0)	281 528.8	337	98.7	3.42 (3.07-3.80)
Lumpectomy plus radiation	63 827 (67.0)	549 816.3	496	176.6	2.81 (2.55-3.04)
Unilateral mastectomy^a^	17 313 (46.3)	163 252.8	238	57.8	4.12 (3.59-4.67)
Bilateral mastectomy^a^	4328 (11.6)	34 917.5	37	8.9	4.14 (2.83-5.49)
Mastectomy, laterality not specified^a^	15 720 (42.1)	126 053.3	128	33.8	3.79 (3.11-4.46)

Abbreviations: ER, estrogen receptor; PR, progesterone receptor; SMR, standardized mortality ratio.

^a^ No radiation therapy.

The SMR for women younger than 40 years was 11.95 (95% CI, 9.66-14.39), for women aged 40 to 49 years was 4.15 (95% CI, 3.73-4.59), for women aged 50 to 59 years was 2.82 (95% CI, 2.54-3.09), for women aged 60 to 69 years was 2.65 (95% CI, 2.39-2.92), and for women aged 70 to 79 years was 3.72 (95% CI, 3.35-4.11) (Table 2). The SMR for White women was 3.03 (95% CI, 2.86-3.21), for Black women was 7.56 (95% CI, 6.76-8.42), for East Asian women was 1.89 (95% CI, 1.36-2.49), and for Southeast Asian women was 2.40 (95% CI, 1.65-3.31). For Black women diagnosed before age 50 years, the SMR was 12.10 (95% CI, 9.94-14.54), and the SMR for White women diagnosed before age 50 years was 4.21 (95% CI, 3.72-4.76).

All women with DCIS underwent surgical treatment, and 68 118 women (47.1%) also received radiotherapy. Among women not treated with radiotherapy, the SMR was 4.12 (95% CI, 3.59-4.67) for those treated with unilateral mastectomy and 4.14 (95% CI, 2.83 to 5.59) for those treated with bilateral mastectomy (Table 2). Among women who underwent lumpectomy, the SMR was 2.81 (95% CI, 2.55 to 3.04) for women treated with radiotherapy and 3.42 (95% CI, 3.07 to 3.80) for those who underwent surgical treatment alone.

There were 1540 women who died of breast cancer in the cohort. Of these, 703 (45.7%) experienced an ipsilateral invasive recurrence or contralateral invasive breast cancer in the interval between DCIS and death from breast cancer. Among women who died, 428 (27.8%) were known to have undergone a mastectomy.

**Figure 1.**
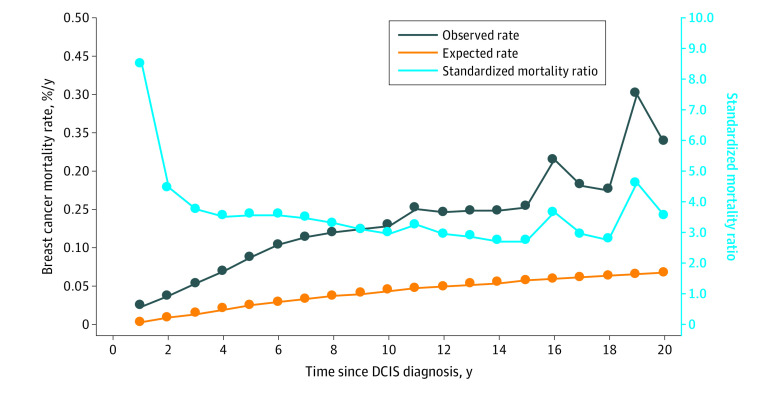
Observed and Expected Annual Risks of Death and Annual Standardized Mortality Ratio From Breast Cancer Following a Diagnosis of Ductal Carcinoma In Situ (DCIS)

**Figure 2.**
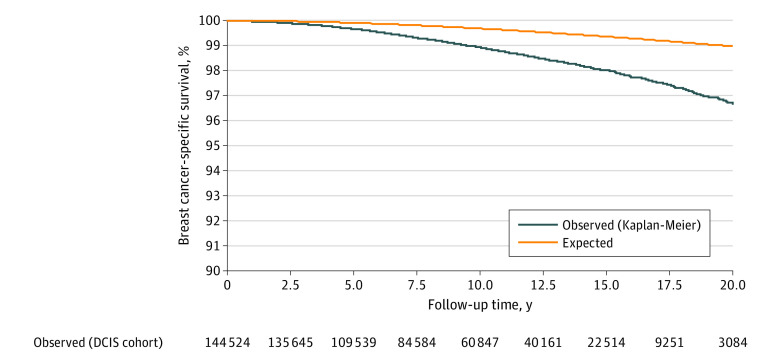
Breast Cancer–Specific Survival Following Ductal Carcinoma In Situ (DCIS)

The annual mortality rate from breast cancer over the entire follow-up period was 0.12% per year. The mortality rate increased for the first 10 years of the follow-up period and was sustained through years 15 through 20 (Figure 1). The cumulative 20-year risk of breast cancer–specific mortality following DCIS was 3.3% (95% CI, 3.1%-3.7%) (Figure 2). For Black women diagnosed before age 50 years, the 20-year risk of breast cancer–specific mortality was 8.1% (95% CI, 5.7%-11.4%).

## Discussion

In this cohort study, we found that the risk of dying of breast cancer in the 20-year period following a diagnosis of DCIS was approximately 3-fold greater than that of a woman of the same age who did not have breast cancer. Our SMR reflects the relative risk of dying of breast cancer in the 20-year period following the diagnosis of DCIS and may not be an accurate reflection of the lifetime increase in the risk of dying of breast cancer after DCIS. Ideally, we would follow all the patients in the cohort until age 80 years to get the most accurate SMR, but we had limited follow-up, and most of the patients were younger than age 80 years in 2016. The mean age at diagnosis in the cohort was 57.4 years, and the mean follow-up time was 9.2 years. It may be that the risk of death from breast cancer tapers off with time from diagnosis. If so, the SMR reported here should not be extrapolated beyond the actual period of follow-up. However, as is seen in Figure 1, the annual mortality rate increased continuously in the first decade after diagnosis and showed no sign of declining in the second decade. It will be of interest to revisit the cohort in a decade to establish the longer-term risks.

Using an earlier version of the SEER DCIS cohort, we previously reported a relative risk of dying of breast cancer of 1.8 for DCIS patients (95% CI, 1.7-1.9), compared with the general population.^3^ While this number is accurate in the appropriate context, in the earlier study, we underestimated the SMR because women in the comparison group may have had breast cancer diagnosed prior to the age at which DCIS was diagnosed in the exposed women. In this study, we restricted our comparison group to women who were cancer-free at the age of diagnosis for women in the DCIS group; consequently, there should be fewer deaths from breast cancer in the controls in this study than in our previous study. This study design allows for a better representation of the impact of a new diagnosis of DCIS on subsequent death from breast cancer. A third approach would be to compare the risk of death from breast cancer in women with DCIS with the risk in women of the same age after a screening mammogram with negative results. In this study, the control arm was derived from observed breast cancer incidence and case-fatality rates, and we do not have screening histories. In this sense, the SMR for death from breast cancer in a woman with screening-detected DCIS is an underestimate of the impact of having screening-detected DCIS, compared with having a mammogram with negative results. Several other studies have also reported an increased risk of breast cancer death following a diagnosis of DCIS compared with the risk in the general population.^4,5,6^ In 2000, Ernster et al^4^ reported a 2-fold increased risk of dying of breast cancer for patients with DCIS diagnosed from 1984 to 1989 in the SEER registry, compared with the general population (10-year SMR, 1.9; 95% CI, 1.5-2.3). In the Swedish Cancer Registry,^5^ patients with DCIS diagnosed from 2000 to 2011 were 3-fold as likely to die of breast cancer as women in the general population (SMR, 3.03; 95% CI, 2.35-3.91). In the Netherlands Cancer Registry,^6^ patients with DCIS diagnosed from 1989 to 2004 (followed for median of 9.8 years) were 3-fold as likely to die of breast cancer than women in the general population (SMR, 3.33; 95% CI, 2.95-3.74). In a subgroup analysis of patients who did not develop a subsequent invasive breast cancer in the follow-up period (ipsilateral or contralateral), the risk of dying from breast cancer after DCIS was still 2-fold that of the general population (SMR, 2.02; 95% CI, 1.89-2.15). The SMRs reported in these studies are comparable to those reported in this study; however, they are also likely to be underestimates of the mortality ratios because the comparison group were all women (using national mortality statistics) rather than women who were initially cancer-free.

To our knowledge, to date there is no empirical evidence that surgical treatment of DCIS reduces the subsequent mortality from breast cancer. Studies that offer a watch and wait approach, such as the Comparison of Operative versus Monitoring and Endocrine Therapy trial,^7^ focus on patients with low-risk DCIS and have too few patients to evaluate mortality differences between those who undergo surgical treatment and those who do not. Given the similar SMRs for women with mastectomy and those with lumpectomy, it is possible that the mortality for women who had neither form of surgical treatment would be similar to these.

In this cohort study, breast cancer death was the principal end point. We did not include invasive recurrence as a primary end point because this information is not necessary to estimate the SMRs. Moreover, we are more confident about the diagnosis of DCIS and the date and cause of death in the SEER database than we are about local recurrences and distant recurrences that were not recorded. Furthermore, some of the deaths might have been from contralateral breast cancer, but the SMR associated with unilateral and bilateral mastectomy were similar.

This study is not designed to generate information on clinical managements of DCIS. The lifetime risk of death following DCIS is approximately 3%, and this level of risk is too low to recommend chemotherapy. There were some women (ie, those younger that 40 years and/or Black) for whom the mortality rate approached 10%, and at this level, chemotherapy might be considered. Ideally, we would be able to identify the few women with DCIS with metastatic potential from the outset and offer them systemic therapy. The current approach is to identify women with a high risk of local recurrence and treat them with radiotherapy initially and with chemotherapy at time of invasive cancer according to the clinical profile. Given that not all women who die of cancer following DCIS experience a local recurrence, the impact of this approach is necessarily limited. It is challenging to identify patients with DCIS who are at high risk of dying; it might be possible to address this question using a case-control approach and compare pathological specimens and molecular expression and other demographic criteria for those who died and those who survived.

### Limitations

This study has some limitations. Treatment was not assigned at random, and we report associations but cannot make causal inferences based on observational data. All patients in this study underwent surgical treatment. We did not attempt to compare the relative benefits of various forms of surgical treatment in this cohort—to do so would have required fine matching, and we have done this previously.^8^ The difference in the SMR between mastectomy and lumpectomy should not be interpreted in favor for lumpectomy. In general, mastectomy patients are younger and have more extensive disease.^8^ Among those with mastectomy, the risk of death was similar for women with bilateral mastectomy and unilateral mastectomy. From this, we interpret that incident contralateral cancers were unlikely to contribute in large part to the overall number of deaths from breast cancer.

It may be that treatments have improved in the past decade and that in the future, mortality rates will be lower than the rates observed in this study. Unfortunately, the use of tamoxifen and other antihormonal therapies is not recorded in SEER. Also, we did not include an untreated group of patients with DCIS for comparison.

We excluded women with microinvasion from the study cohort (7.7% of all DCIS cases in SEER have microinvasion recorded).^9^ There may be some residual misclassification, such that patients who died of breast cancer may have had an overrepresentation of microinvasion, but we have used the standard definitions and reporting practices available through SEER.

## Conclusions

This cohort study found that women with DCIS had a 3-fold increased risk of death from breast cancer after surgical treatment. The SMR was lower among women who received lumpectomy plus radiation compared with women who received lumpectomy alone. The rate of breast cancer death was nearly 12-fold higher among women diagnosed with DCIS before age 40 years and 7-fold higher in Black women diagnosed with DCIS compared with the general population.
